# Predicting neuronal dynamics with a delayed gain control model

**DOI:** 10.1371/journal.pcbi.1007484

**Published:** 2019-11-20

**Authors:** Jingyang Zhou, Noah C. Benson, Kendrick Kay, Jonathan Winawer

**Affiliations:** 1 Department of Psychology, New York University, New York City, New York, United States of America; 2 Center for Magnetic Resonance Research, Department of Radiology, University of Minnesota, Twin Cities, Minnesota, United States of America; 3 Center for Neural Science, New York University, New York City, New York, United States of America; 4 Stanford Human Intracranial Cognitive Electrophysiology Program (SHICEP), Palo Alto, California, United States of America; University of Texas at Austin, UNITED STATES

## Abstract

Visual neurons respond to static images with specific dynamics: neuronal responses sum sub-additively over time, reduce in amplitude with repeated or sustained stimuli (neuronal adaptation), and are slower at low stimulus contrast. Here, we propose a simple model that predicts these seemingly disparate response patterns observed in a diverse set of measurements–intracranial electrodes in patients, fMRI, and macaque single unit spiking. The model takes a time-varying contrast time course of a stimulus as input, and produces predicted neuronal dynamics as output. Model computation consists of linear filtering, expansive exponentiation, and a divisive gain control. The gain control signal relates to but is slower than the linear signal, and this delay is critical in giving rise to predictions matched to the observed dynamics. Our model is simpler than previously proposed related models, and fitting the model to intracranial EEG data uncovers two regularities across human visual field maps: estimated linear filters (temporal receptive fields) systematically differ across and within visual field maps, and later areas exhibit more rapid and substantial gain control. The model is further generalizable to account for dynamics of contrast-dependent spike rates in macaque V1, and amplitudes of fMRI BOLD in human V1.

## Author summary

This paper contributes to modeling and understanding the neuronal dynamics of visual cortex in four ways. First, we proposed a model that describes stimulus-driven neuronal dynamics in a simple and intuitive way. Second, we applied the model to intracranial EEG data and found regularities of response dynamics across and within human visual field maps. Third, the model was generalizable across different ways of measuring brain activity, allowing us to potentially link the sources underlying diverse measurements. Fourth, we comprehensively summarized existing models of neuronal dynamics, and identified effective components that give rise to accurate prediction.

## Introduction

**Fig 1:**
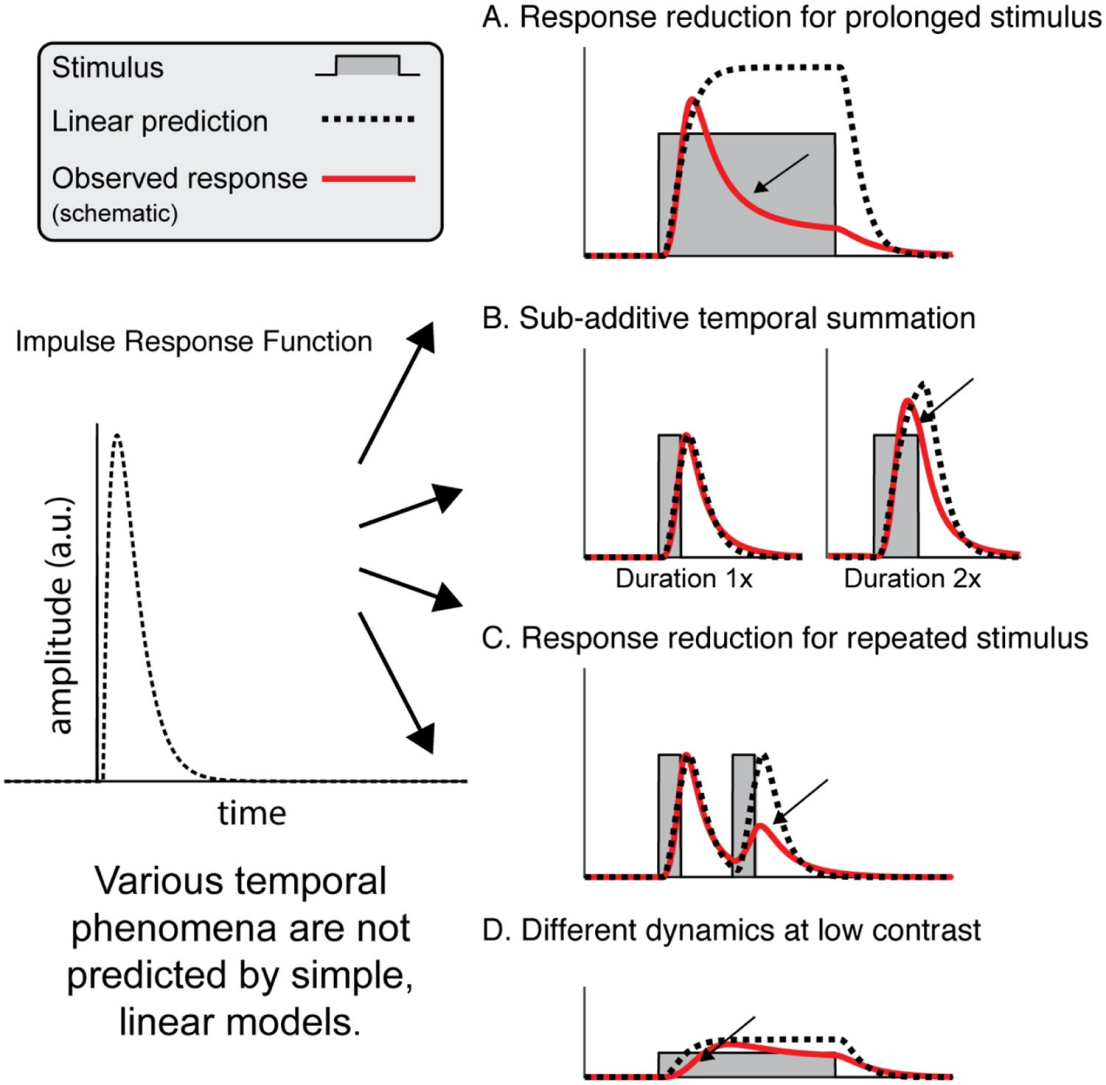
Schematic of different temporal phenomena observed in neural response time courses. For each phenomenon, we show a schematic with a stimulus time course (gray shading), a linear prediction (black dashed line), and a cartoon illustration of plausible neuronal responses consistent with prior findings (red line). The linear prediction is the result of convolving an impulse response (left) with a stimulus time course. A. For a sustained stimulus, the neuronal response reduces after an initial transient, differing from the sustained linear prediction [e.g., 2, 3]. B. Neuronal responses sum sub-linearly in time: doubling the stimulus duration results in a total response that is less than double (less than the linear prediction) [3, 4]. C. For two presentations of a single image with a brief gap in between, the neuronal response to the second presentation is lower than the linear prediction (e.g., refs [2, 4, 5]). D. Compared to the linear prediction, the neuronal response to a low contrast stimulus is both lower in amplitude and delayed [4, 6, 7].

Our visual system extracts behaviorally relevant information from a large quantity of inputs spread over space and time. To do so, some aspects of visual inputs are prioritized over others. In space, for example, the center-surround receptive fields in retinal ganglion cells enhance sensitivity to contrast, while attenuating sensitivity to diffuse illumination [1]. Over time, some aspects of visual inputs are also prioritized over others. First, the neuronal response such as the time-varying spike rate (peristimulus time histogram, ‘PSTH’) to a sustained stimulus gradually declines following an initial transient [e.g., 2, 3] (Fig 1A). Second, responses to longer stimulation are less than the linearly predicted response (sum of shifted copies) from briefer stimulation [3, 4] (Fig 1B). Third, when two stimuli are presented close in time, the response to the second stimulus is reduced compared to the first [2, 4, 5] (Fig 1C). Fourth, dynamics of neuronal responses depend on stimulus contrast–the response to low contrast stimulation is delayed and reduced in amplitude, compared to high contrast stimulation [4, 6, 7] (Fig 1D). These phenomena are consistent with new and more reliable information (e.g. higher contrast) being prioritized in visual processing.

To achieve a unified understanding of these seemingly disparate phenomena, here, we developed a general yet simple model that predicts neuronal dynamics in response to a static image whose contrast varies arbitrarily over time. The model is based on canonical neuronal computations [8, 9], and is related to, but is easier to compute than, previously proposed models that capture some [10, 11] or all [11–13] the phenomena summarized above. We obtained novel insights into visual processing by applying the model to neuronal data. Fitting the model to intracranial EEG data revealed differences in neuronal dynamics between posterior and anterior visual field maps, and across eccentricities within a single map. We further generalized the model to make predictions for macaque V1 spiking data in response to a static grating of different contrasts, and fMRI BOLD amplitudes in human V1 in response to static images with different time courses.

## Results

### Delayed normalization model: Form and predictions

The delayed normalization model has an LNG structure (Linear, Nonlinear, Gain control). In the linear stage, the model convolves the contrast time course of a single image with an impulse response. The output of this linear computation is then full-wave rectified (absolute valued) and expansively exponentiated. The interpretation of the full-wave rectification depends on the type of measurement. In single-cell measurements, full-wave rectification can be interpreted as the result of a linear combination of half-wave rectified (zeroing negative linear predictions) responses from two cells with complementary receptive fields–the excitatory part of one cell’s receptive field corresponds to the inhibitory part of the other [14]. This assumption is not physiologically realistic over the entire range of all possible stimuli, because the linear-rectified assumption of the upstream neuronal computation only holds, in general, for small stimulus ranges. For the population response, we assumed the same model form as for the single cell’s response here. The rectified response is then exponentiated, and the exponentiation step approximates the non-linear transform from membrane voltage to spiking. The exponential constant is empirically estimated, and is likely to be expansive (exponent > 1) as found in previous estimates in single cells and in cell populations [8, 15]. There are multiple synapses between retinal ganglion cells and cortical neurons, which could in principle be modeled as a cascade of linear-nonlinear operations. (We return to this in the Discussion). Instead, we used a single exponentiation to model multiple stages of voltage to spiking transform, as cascaded point-wise exponentiations can be reduced to a single exponentiation, or, (*x*^*a*^)^*b*^ = *x*^*c*^ for *c* = *ab*.

The last and the most important computation of the model is a delayed gain control, implemented as a divisive normalization. The numerator here is the exponentially rectified linear output. The denominator consists of two exponentiated components: a constant (semi-saturation) and a low-passed rectified-linear output. The low-pass causal filter (implemented as an exponential decay) in the denominator is what gives rise to the predicted adaptation behavior. Intuitively, at stimulus onset, the linear filter sums the stimulus contrast within some time window and the numerator dominates the predicted initial response, resulting in a sharp rise in the response. The response then starts to decay once the sluggish gain control kicks in and starts to dominate. A history-dependent normalization signal has been proposed, and implemented as part of a feedback circuit [14, 15] to describe how steady state normalization could arise as an equilibrium point of a dynamical feedback system. Here, we provided an alternative and possibly simpler implementation of the same process. Because delayed gain control is essential for the desired model behavior, we refer to the model as a delayed normalization (DN) model.

**Fig 2:**
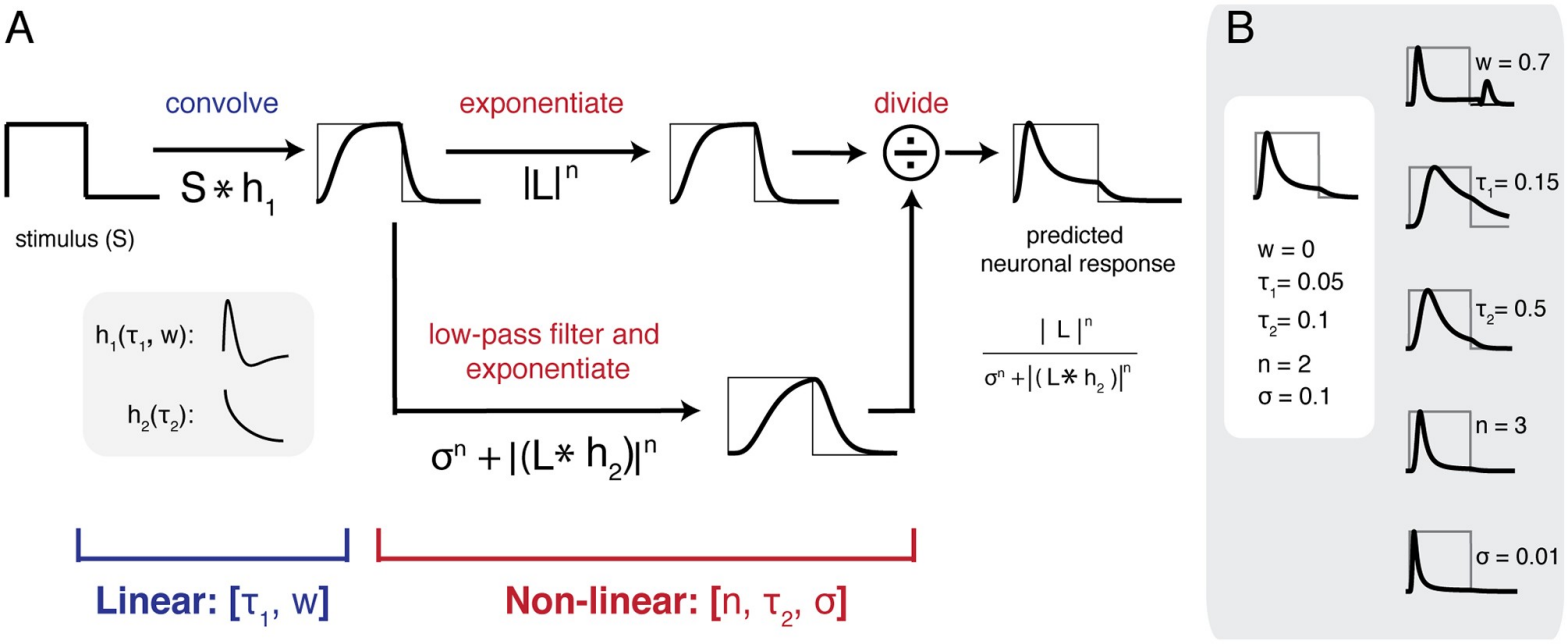
The delayed normalization (DN) model. *(A)* The input to the model is the contrast time course of a stimulus, *S*, which is 0 when the stimulus is absent and 1 when it is present. First, the model computes the linear neuronal response by convolving *S* with an impulse response function *h*_*1*_ (parameterized by τ_1_ and *w*). The linear output is then full-wave rectified and exponentiated by *n*. We assumed *n*>1 in this paper. The exponentiated output is divisively normalized by a denominator that consists of two components: a semi-saturation constant (σ), and a causally low-pass filtered version of the driving signal. Both components were raised to the same power *n*. The predicted neuronal response (right) to the example input stimulus *S* (left) includes a transient followed by a lower-level, more sustained response. (B) The effects of varying each of the 5 parameters are shown. For example, larger *w* means a more biphasic impulse response, therefore a larger transient response at stimulus offset (top row). In all simulations, the default parameters are *w* = 0, τ_1_ = 0.05, τ_2_ = 0.1, n = 2, σ = 1. For more details of model behavior see S1 Fig.

The DN model was parameterized by five variables: τ_1_, τ_2_, *w*, *n* and σ. For the linear computation, we implemented a biphasic impulse response function (IRF) as a weighted difference between two monophasic response functions. These were modeled as gamma functions, parameterized with time constant τ_1_ for the positive function and 1.5τ_1_ for the negative function. The weight (*w*) was applied to the second (negative) function and has value between 0 and 1. Weight 1 means the IRF is maximally biphasic (for *w* within [0, 1]), and the model predicts a large transient response at both stimulus offset and onset, and predicts 0 for a constant stimulus at steady state. Weight 0 means the IRF is monophasic, and the model predicts a transient response at stimulus onset only, and a positive response in steady state for a constant stimulus (Fig 2B, S1 Fig). When fitting the DN model to time-resolved data, unless specified otherwise, we fix *w* to be 0 in order to reduce the number of free parameters, and because the offset transient response is small in most data. The only exception is in peripheral visual field maps, where offset transients were large, therefore we allow *w* to vary when fitting the model to data binned over eccentricities. The second variable that parameterizes the linear computation is a time constant τ_1_, the time to peak in the first pulse of the IRF. τ_1_ controls the width of the impulse response, or the length of temporal summation in the DN model. For predictions to sustained stimuli, τ_1_ contributes to the width of the initial transient response (see Fig 2B and S1 Fig row 1).

The remaining three variables parameterize the history-dependent or delayed divisive normalization. The numerator of the normalization is a linear response raised point-wise to a power *n* (*n*>0). The denominator has two terms, a semi-saturation constant σ (raised to the same power *n*) to prevent the model from being ill-defined (when linear response is 0 at a time point), and a delayed and low-passed driving signal (linear-rectified output). The causal low-pass filter was implemented as an exponential decay, parameterized by a time constant τ_2_. For predictions to sustained stimuli, a larger τ_2_ results in a smoother decay from the transient. The decay is smoother because the normalization signal is averaged over a longer response history (Fig 2B and S1 Fig row 2). The low-passed output is then raised to a power *n*. Given a single level of stimulus contrast, the role of *n* and σ are largely shared: large *n*, as well as small σ, predicts a sharp transient decay. Yet we include both parameters in the model construct because the two parameters predict distinct dynamics at different stimulus contrasts: varying σ predicts varying response dynamics (e.g. response time to peak) at low contrast, whereas adjusting *n* scales the response level with different stimulus contrast only (Fig 2B and S1 Fig row 3&4).

We assume a self-normalization process in the DN model, i.e. the numerator and the denominator share the same driving signal/linear response (‘L’ in Fig 2A). Self-normalization is sufficient to account for our current available data. It would be natural to generalize the model to include distinct time courses for driving (numerator) and normalizing signals (denominator). For example, when we believe that the normalizing signal is computed over a large neuronal population, the time course of which is distinct from a single neuron’s driving signal.

Following stimulus onset, the DN prediction increases rapidly due to convolution and exponentiation, and then reduces due to normalization, remaining at a lower, sustained level until stimulus offset. Although summation (convolution) and neuronal adaptation (normalization) both occur continuously throughout the predicted time course, different parts of the time course emphasize different neuronal phenomena: The initial response increase primarily reflects temporal summation (combining current inputs with past inputs), whereas the reduction following initial transient reflects adaptation, since the response level declines when the stimulus is unchanging.

In the remaining parts of the Results, we used data from different measurement techniques to examine the 4 temporal phenomena shown in Fig 1: reduced responses for prolonged stimuli (ECoG), sub-additive temporal summation (fMRI), reduced responses for repeated stimuli (fMRI), and delayed response at low contrast (single unit spike rates). Because the stimuli differed across experiments, different datasets exhibit different phenomena (for example, the BOLD experiment varied the stimulus duration but not the contrast, and the single unit experiments varied the contrast but not duration).

### Phenomenon 1: Response reduction for prolonged stimuli

In this section, we show that the DN model captures the transient-decay neuronal dynamics at sustained (hundreds of milliseconds) presentation of static images (Fig 1A). Moreover, details of the transient-decay pattern differ across cortical locations, and this difference was reflected in DN model parameters.

#### Differences along the visual hierarchy

**Fig 3:**
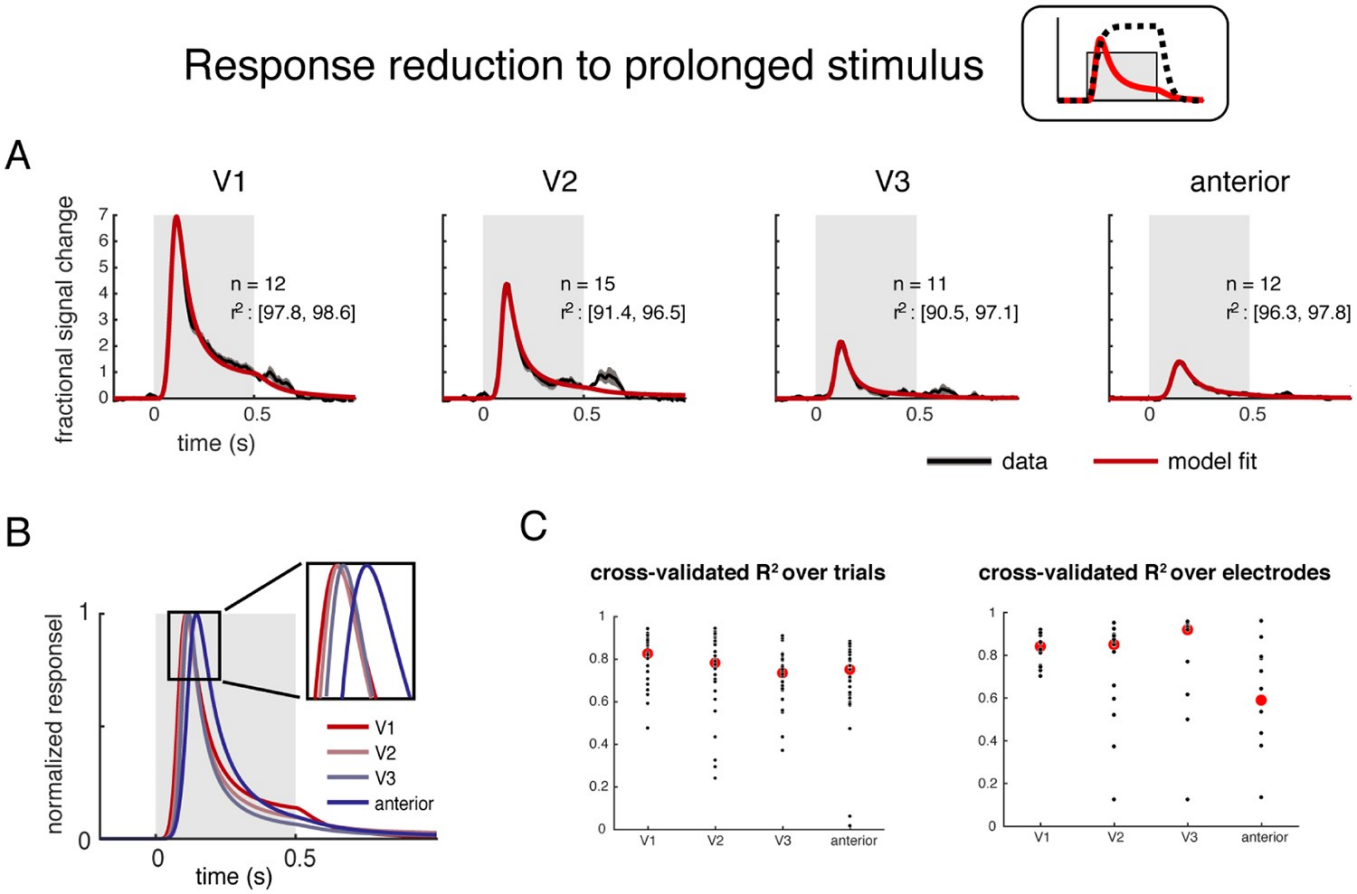
The DN model captures the response reduction for prolonged stimuli at different cortical locations. (A) The DN model fits (red) accurately describe the ECoG broadband time course (black) in multiple ROIs. Data were averaged across trials and electrodes within ROIs, and models were fit to the average time course. Each trial had a 500-ms stimulus (gray box) followed by a 500-ms blank. Plots show the mean and 50% CI for data (bootstrapped 100 times across electrodes within an ROI), and the model fit averaged across the 100 bootstraps. The number of electrodes per ROI and the 50% CI of model accuracy (r^2^ per bootstrap) are indicated in each subplot. (B) The model fits for the 4 ROIs are plotted together, scaled to unit height. For this plot, the latency was assumed to be 0 for each ROI, so that the difference in time to peak reflects a difference in integration time rather than a difference in response latency. (C) Cross-validation over trials and over electrodes. 30-fold leave-one-out cross validation over trials was performed on the 30 repeats. Red dots represent the median r^2^ across trials, and black dots are the leave-one-out prediction to each trial. Leave-one-out cross validation was also performed over electrodes. Details of the cross-validated fit were presented in S3 Fig.

We extracted part of the intracranial EEG (or ECoG/electrocorticography) signal that is thought to be correlated with the average local neuronal firing rates for model fitting. To do so, we computed the envelope of the high frequency (70–210 Hz, ‘broadband’) time courses from a large set of human ECoG electrodes spanning multiple visual field maps. Spectral patterns of the responses in these electrodes (but not the time courses) were analyzed for a prior publication [16]. Based on the estimated cortical locations (population receptive field center) of the electrodes from a separate retinotopy experiment, we binned these electrodes into four ROIs (V1, V2, V3, and anterior maps). The “anterior maps” ROI (Fig 3) includes electrodes from ventral (hV4, VO-1/2), lateral (LO-1/2), and dorsal (V3A/B, IPS) visual field maps. They were binned into one ROI to match the number of electrodes in the V1-V3 ROIs (n = 12, 15, 11, 12; V1, V2, V3, anterior).

In each trial during the experiment, a static texture (22°-diameter) was presented for 500 ms followed by a 500-ms blank. The textures were noise patterns with 1/*f*^*n*^ amplitude spectra, and *n* = 0, 1, or 2 (white, pink, or brown noise). The experiment also included large field grating stimuli, and responses to these stimuli were not included for analysis because they elicit unusual time courses (large, narrowband gamma oscillations). We averaged the broadband time series across stimulus classes, trials, and electrodes within each ROI before fitting the average time series with the DN model. (See S2 Fig for individual electrode locations and responses).

The DN model provided good fit to the broadband time course from all 4 ROIs, with the variance explained by the model between 90% and 99%, and cross-validated variance explained generally above 70%, especially across trials. The responses in each of the 4 ROIs exhibited the characteristic pattern whereby the amplitude substantially declined following an initial large response (e.g., as depicted in the schematic in Fig 1A). The largest amplitude responses were in the earliest areas: from 7-fold over baseline in V1 to ~1.5-fold in the anterior maps. In addition to amplitude differences, there were also quantitative differences in the shape of the time courses from different ROIs. These differences were reflected in both the model predictions and in summary metrics derived from the model fit (Fig 3, right panel). For a similar result from another subject, see S4 Fig.

**Fig 4:**
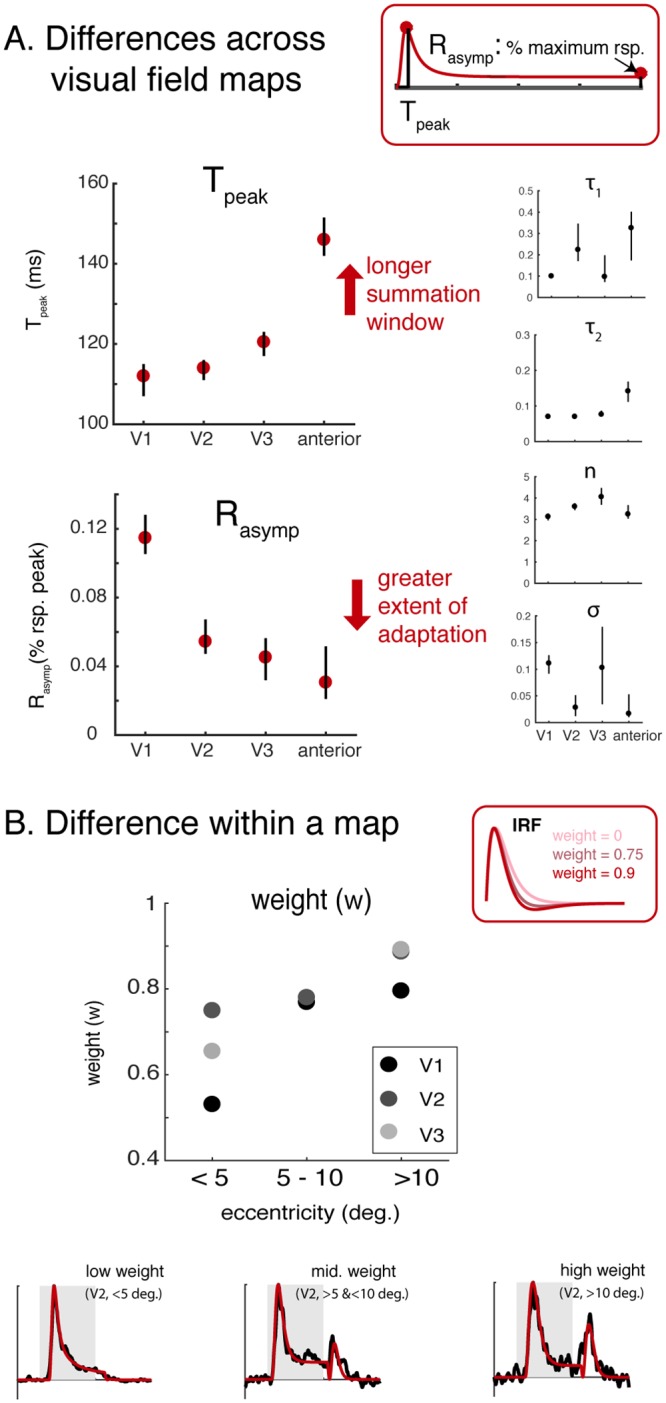
The DN model captures difference of temporal dynamics at different cortical locations. (A) Temporal summation window length and the extent of gain control increase along the visual hierarchy. The model parameters fit to the data are shown on the right. The model fits were then summarized by two metrics. Tpeak is the duration from the onset of a sustained stimulus to the peak response, excluding the onset latency. Tpeak is longer for later ROIs, ranging from ~115 ms (V1) to ~145 ms (anterior ROIs). Rasymp is the level at which the response asymptotes for a sustained stimulus, as a fraction of the peak response. A smaller Rasymp indicates a greater extent of gain control. Rasymp is largest in V1 (~0.12) and declines in extrastriate areas. See S5 Fig for individual electrode results. *(B) Offset response as a function of eccentricity*. The lower plots show the time series and model fits to 3 example electrodes. The offset response increases from fovea to periphery. This pattern holds across all 3 ROIs, as shown in the dot plot. Each dot is the mean weight (*w*) on the negative lobe of the biphasic response. Larger values of *w* predict larger offset responses.

We derived two interpretable summary metrics to quantify model behavior in each ROI (Fig 4A): time to peak (Tpeak) and asymptotic response amplitude (Rasymp). Each metric quantifies some aspect of the model response to a sustained stimulus. Tpeak is the time to peak predicted by the model for a sustained stimulus. It indicates temporal summation window length. Tpeak was shortest in V1 and V2 (120-125ms), and longer in the more anterior areas (~145 ms). This summary metric excludes an onset latency, which was fit as a nuisance parameter, and hence a longer Tpeak reflects a longer summation window, not a longer latency to respond. Rasymp is the ratio between the peak and the sustained amplitude. A low Rasymp indicates a larger extent of normalization. Rasymp was the highest in V1 (therefore the least amount of gain control up to V1), and decreased substantially in extrastriate areas, paralleling previously observed non-linearities in spatial summation across visual areas [17]. We summarized the differences between ROIs using these derived metrics instead of using the DN model parameters because the relationship between a single model parameter and the model output tends not to be straightforward. For example, either increasing *n* or decreasing σ leads to a decreased sustained response, as shown in Fig 2B, hence neither parameter alone sufficiently represents the amount of gain control estimated from the data. Although these separate model parameters are less easily interpretable, they tend to show some of the same patterns as the summary metrics: shortest time constants in V1 and longest in the anterior maps.

#### Differences as a function of eccentricity

Previous work has shown that within V1, regions with more peripheral eccentricities are more sensitive to visual transients [11]. Inspection of our data in V1-V3 agrees with this pattern, as some electrodes with more peripheral receptive fields show a small positive deflection 100–200 ms after stimulus offset. This offset transient was not salient in the mean time-series across electrodes (Fig 3), but it was clear in some individual electrodes (Fig 4B, S5 Fig).

To quantify the offset transient response, we fit the DN model with varying *w* (weight of the negative gamma function in the IRF) to the time course within each individual electrode. For tractability of the model fit, we fixed the exponent parameter *n* at 2. For each visual map, we separated the electrodes into three electricity bins (<5, 5–10, and >10 degrees) and averaged the parameters fitted to individual electrodes within a bin.

The model provided excellent fits to the full time-course of the response in individual electrodes including stimulus offset (S5 Fig). For V1, V2, and V3, electrodes with peripheral population receptive field (pRF) centers had higher weights (~0.8, >10 degrees) on the negative lobe of the impulse response function compared to foveal electrodes (~0.5, 0–5 degrees), consistent with the fMRI studies showing that more peripheral locations (within visual field maps) are more sensitive to stimulus onset and offset transients [11, 18]. We did not perform this analysis for the more anterior areas due to an insufficient number of electrodes within a visual field map and therefore within an eccentricity bin.

#### Generalization across instruments

Above, we showed that the DN model accurately fit the ECoG broadband time series from different visual areas and different eccentricities. Here, to test generalizability, we fit the model to example time courses from 3 measurement types in early visual cortex obtained from prior publications (S4A Fig). Each time course was the response to a static contrast pattern viewed for a few hundred ms: (1) single neuron PSTH from macaque V1 [6]; (2) multiunit spike rates (by taking the envelope of the band-pass filtered raw signal between 500 and 5k Hz, see Method) from depth recordings in human V2/V3 [19]; and (3) LFP from the same depth recordings in human V2/V3 [19]. The time courses of the 3 measurements, although differing in detail, have a common pattern: there is a large, initial transient response after stimulus onset, followed by a reduction to a lower, more sustained response. This pattern was accurately fit by the DN model prediction, explaining 93% to 99% of the variance in the 3 responses. This transient/sustained pattern in these example time courses is similar to that observed in many other electrophysiological studies [e.g., 2, 3, 20].

### Phenomena 2&3: Sub-additive temporal summation and reduced responses for repeated stimuli

In our prior fMRI studies [4], we fit a static normalization model to fMRI BOLD amplitudes in response to one- and two-pulse stimuli of various durations and inter-stimulus intervals. Responses to the one-pulse stimuli of different durations demonstrated sub-linear temporal summation (schematic in Fig 1B), and responses to the two-pulse stimuli were consistent with reduced responses for repeated stimuli (Fig 1C). Here, we asked whether the same model fit to ECoG data (previous section), with the same parameters, can accurately predict the previously published fMRI responses, thereby accounting for these two sub-additive temporal phenomena.

**Fig 5:**
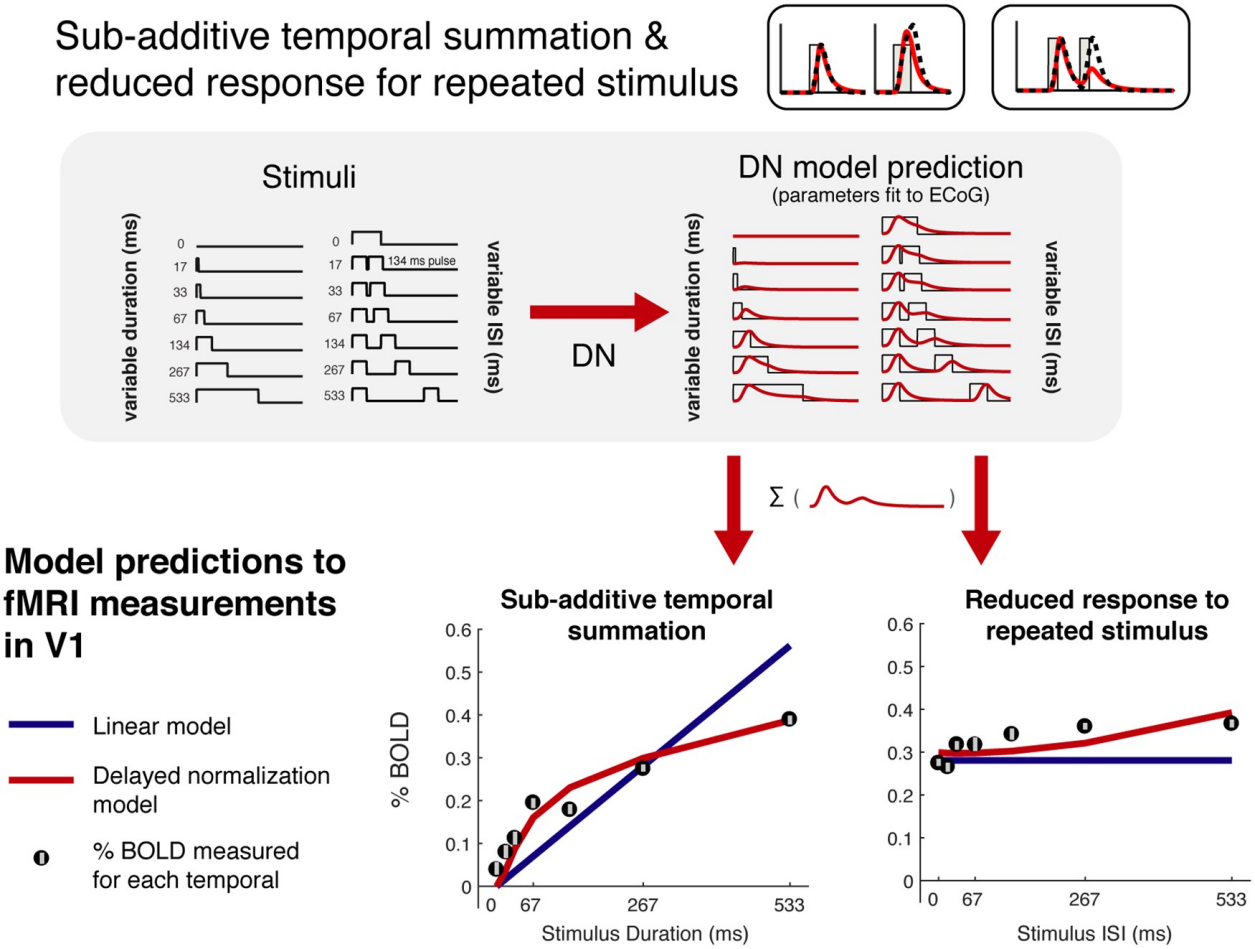
DN model captures sub-additive temporal summation and adaptation. There are two types of temporal profiles used for the fMRI experiment: one-pulse stimuli with varying durations and two-pulse stimuli (134 ms each) with varying ISI. To generate DN model predictions to these stimuli, we used the median DN parameters fit to the V1 broadband time course measured in individual electrodes (S3 Fig). To convert the prediction to percent BOLD, we summed the predicted time course for each temporal profile and fit a single gain factor to minimize the difference between the predictions and the fMRI data. The DN model predictions (red) better capture the BOLD data than the linear prediction (green) (r^2^: 0.94 vs. 0.81).

In brief, the fMRI subjects were presented with a large-field contrast pattern either once or twice per 4.5-s trial (Fig 5, top). For single-pulse trials, the stimulus duration varied from 0 (i.e., no stimulus) to 533 ms. For double pulses, the image was viewed twice for 134 ms each, with an inter-stimulus interval spanning 0 to 533 ms. In the previous section, we fit the model to ECoG data that has millisecond resolution, whereas fMRI measures brain activity about every second or two. Hence to make the ECoG predictions commensurate to the fMRI measures, we did the following.

First, we generated the predicted time-varying neuronal response for each stimulus within each of the 4 ROIs using parameters estimated from the ECoG experiment. Because we had more electrodes than ROIs, within each ROI we used the median model parameters across ECoG electrodes (Fig 4A).

Second, we summed this predicted time series to quantify the total predicted neuronal response for that trial. Finally, we scaled the responses in order to translate them into units of percentage BOLD based on a fit of the best single scale factor across all 13 temporal conditions. Overall, this procedure yields one predicted number for each temporal condition per ROI. The experimentally measured fMRI BOLD amplitudes (one per stimulus condition) were derived by solving a general linear model (GLM). Because the GLM already accounts for the hemodynamic response function, the BOLD amplitude predicted from the DN model can be compared directly to the fMRI-measured amplitude. Because the DN model parameters were derived from the ECoG data alone, there were no free parameters other than the gain to convert the summed time series to percent BOLD units. Although the DN models were solved with different participants, different stimuli, and a different instrument, they nonetheless accurately fit the BOLD data (*r*^*2*^ = 94%, Fig 5, bottom). This is more accurate than predictions from a linear model (*r*^*2*^ = 81%).

Both the measured BOLD response and the predictions derived from the ECoG model fits show two patterns consistent with neuronal phenomena schematized in Fig 1. First, the BOLD signal shows evidence of sub-additive temporal summation, in that responses to long-duration stimuli are less than the linear prediction, and responses to short duration stimuli are greater than the linear prediction. This pattern is accurately captured by the DN model derived from ECoG, but not from a linear model without any normalization (compare red versus green line fits in Fig 5, left plot).

Second, the BOLD signal shows evidence of reduced responses for repeated stimuli. This can be seen when the inter-stimulus interval for the two-pulse stimuli is short and the response is low (adaptation), compared to when the interval is longer and the response is higher (recovery from adaptation). This pattern is not predicted by a linear model, for which the total predicted response is the same irrespective of the inter-stimulus interval, but it is predicted by the DN model (compare red versus blue lines in Fig 5, right plot).

### Phenomenon 4: Different dynamics at low contrast, measured using single unit spike rate

In this section, the model was generalized to account for single unit peri-stimulus time histograms (PSTHs) measured in macaque V1 in response to stimuli of variable contrasts. In previous sections, the DN model took a time course of binary values as input, where 1 represented stimulus present and 0 represented stimulus absent (neutral gray screen). Here, the model was generalized to take any value *c* in the range [0, 1] to represent a stimulus of contrast level *c*. We fit the model to PSTHs observed from 3 complex cells previously published in [6].

**Fig 6:**
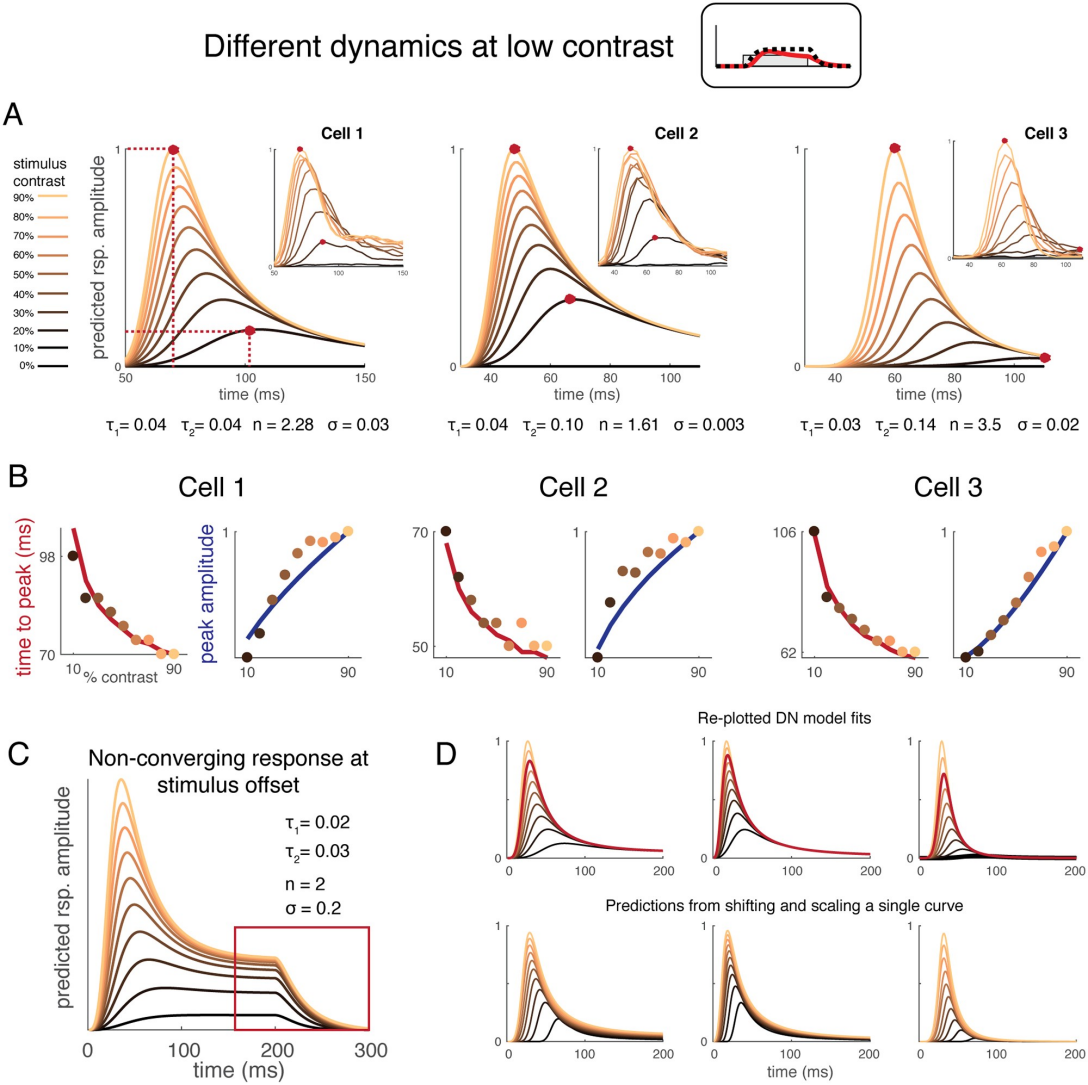
DN model captures delayed responses at low contrast. *(A)* The DN model was fit to single unit spike rate data from macaque V1, with stimulus contrasts ranging from 0% to 90%. The input time course for model fitting was scaled to the stimulus contrast. A single model was fit to all stimuli (10 time-courses) separately for each of the three cells. Model fits are shown in the main plots and data in the insets. The model captures both the lower response amplitudes and slower temporal dynamics at low contrast. Data from [6], provided by W. Geisler. *(B)* Time to peak (ms) and peak amplitude (normalized spike rate) for single unit data as a function of contrast. The 3 cells are those plotted in (A). The data points are the cell responses and the curved lines are the DN model fits. The colors of the dots match the colors in (A), indicating stimulus contrast. *(C)* A set of model parameters that predict non-converging response levels at stimulus offset. *(D)* The top row is identical to the model predictions in panel A except they are shown for an extended period (up to 200 ms). The predicted response to 70% contrast is highlighted, and was used to scale and shift to predict other time courses, as shown in the bottom row.

Although the three complex cells differ in their overall dynamics, within each cell, the PSTH dynamics vary in a consistent way with stimulus contrast: compared to high contrast, response peaks tend to be lower and later at low contrast (Fig 6A). To demonstrate that the model captures these two data features, we simultaneously fitted the model to ten time courses for each cell–each time course corresponds to a PSTH for a static grating presented at a contrast level ranging from 0 to 90% in equal step sizes. The model captures the two data features (Fig 6B), as well as produces high overall prediction accuracy for the time courses: 96%, 97%, 96%, respectively for each cell. The model parameters fit to each set of cell responses are illustrated below each panel in Fig 6A. The parameters are similar in range to those fit to ECoG time courses in V1, but are quite different from the mean of the ECoG V1 values (for example, τ_1_ was ~40 ms for the single cells, but ~100 ms for broadband ECoG V1). There are at least three factors that could contribute to this difference: (1) ECoG measures a large population of cells simultaneously, whereas here, we quantify a single cell’s response at a time, (2) ECoG has a sampling bias towards large pyramidal cells, and (3) there are differences in stimulus patterns used (gratings versus noise textures), site retinotopic coordinates, and species. Future work matching as many of these parameters as possible will help resolve the source of these quantitative differences between our ECoG data and the single unit data.

The model captures low response amplitude at low contrast, because in the divisive normalization equation, although the numerator (LN response) and the normalization pool response (the second component in the denominator) are similar in amplitude, at low contrast, they are both relatively small compared to the semi-saturation term σ (small numbers divided by a small number). At high contrast, the numerator is large compared to σ, so the overall response amplitude is relatively large (large numbers divided by a small number). The model captures the slow response dynamics at low contrast for the following reason: the peak time in the response reflects a tradeoff between summing of the impulse response function (resulting in an amplitude increase) and the normalization extent (resulting in an amplitude decrease). Large normalization at high contrast causes an earlier response decrease, and therefore an earlier peak time. At low contrast, the response increases slowly because the impulse response function sums less contrast per unit time, and the normalization tradeoff occurs later because the normalization extent at each time point also depends on temporal summation.

For all three cells, the model predicts that responses to different stimulus contrasts all converge over the time course of the stimulus presentation. In particular, the model predicts that the response time courses are near identical at all contrasts after stimulus offsets. This convergence feature is predicted by the model, but 1) this is not a consistent feature across the three cells, and 2) the model does not predict this feature in general, given different combinations of the parameters, especially when the extent of normalization is low (small *n* and/or big σ). In Fig 6C, we show a combination of parameters that predicts different falling edges for responses at different contrasts. But in general, the model does not predict a “cross-over”: the falling edge for the response to low contrast is higher at all points than that for high contrast. This cross-over exists in some cells (e.g. cell 3), but whether this feature is a typical feature of cells awaits confirmation from more data. Chalk et al. 2017 [21] examined a two-neuron network, the gain control of which was either implemented as a division or a subtraction [22]. The prediction of the difference between the two gain-control implementations (for excitatory neurons) is that the divisive model predicts the convergence of the falling response edge, whereas the subtraction model predicts parallel edges, as in Fig 6C. Here, the DN model, with different settings of parameters, is capable of capturing both response patterns at stimulus offset.

A descriptive model of different response time courses was proposed by Albrecht et al. 2001 [6]. Their model is not a general input-output model, but does accurately characterize their measured PSTHs. They concluded that PSTH shapes at different contrasts could be approximated as scaled and shifted copies of each other. The DN model reproduces this observation. We show that the response time course at 70% contrast (Fig 6D) can be shifted and scaled to approximate the responses at other contrasts for each cell. The approximation was quite good, with >98% variance explained for each cell. The result holds when using any response time course at or above 50% contrast, with a mean variance explained of at least 95% for all cells. Thus, our model predicts a similar pattern to Albrecht et al., but is more general and flexible as it predicts responses to arbitrary stimulus time courses.

## Discussion

We proposed a model of neuronal dynamics that generalized in 3 ways. First, the model accurately accounted for diverse temporal phenomena, including sub-linear summation, neuronal adaptation, and slower dynamics at low contrast. Second, the model generalized across measurement types, including the fMRI BOLD signal, the ECoG broadband signal, and single unit PSTH, among others. Third, parameters of the model varied systematically both within and across visual field maps.

### The delayed normalization model and related computational frameworks

In our previous work [23], we developed a neuronal model to account for fMRI responses to briefly presented static stimuli (less than 1 second). A limitation of this model primarily lies within the measurement method–fMRI samples coarsely in time, and allows only for tests on the sum, instead of the dynamics, of the time course predictions. Here, we introduced time-resolved ECoG data to constrain model dynamics, and we extended upon the previous model to include a delay computation to the divisive gain control. Our current model (DN) subsumes our previous model: the divisive gain control in DN depends on response history with a time constant τ_2_, and the previous model, with no delay, is equivalent to the the DN model with an infinitesimally small τ_2_, i.e., instantaneous gain control. Different values for τ_2_ produce different prediction dynamics–with a monophasic impulse response function, the DN model predicts a transient-decay response shape to a sustained stimulus, whereas our previous model cannot capture response decay after the initial transient.

**Fig 7:**
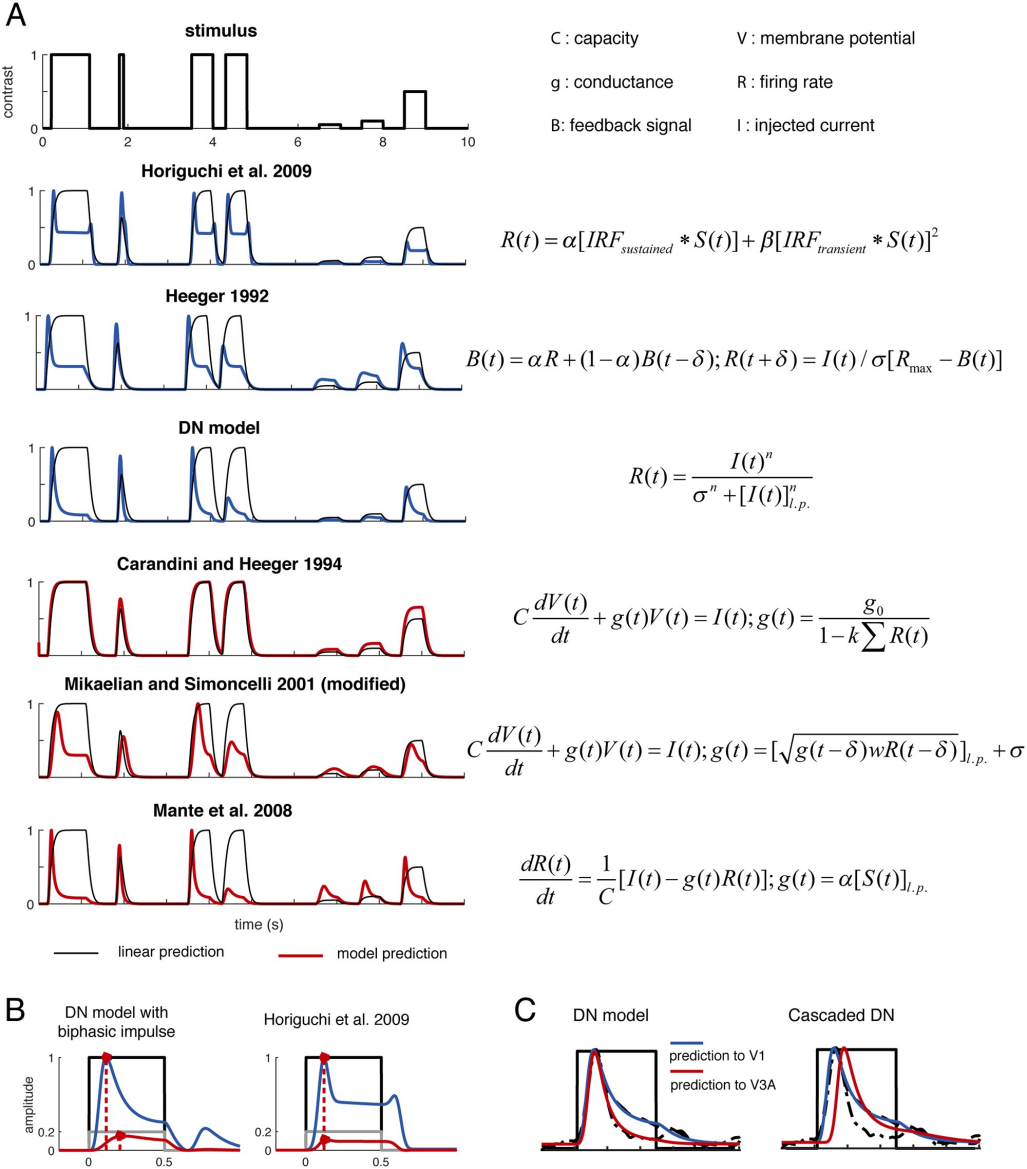
Comparison between temporal models. **A.** We compared two groups of models by simulating their outputs to a set of stimulus time courses (different stimulus durations, ISIs, and different stimulus contrasts). One group of models (red) is inspired by RC-circuit models of normalization, and assumes gain control is implemented by changes in membrane conductance associated with spiking. The other group of models (blue) do not make this assumption. Within each set, there are some (but not all) models that capture the properties of the response time courses summarized in Fig 1. **B.** The two temporal channels model does not assume a gain control component. As a consequence, when stimuli are of lower contrast, the model captures the reduced response amplitude but not the slower dynamics. Within a model group, if the level of gain control depends on the instantaneous instead of the history-dependent response, the model does not reproduce the transient-decay response shape. **C**. The difference between the response time course from a striate versus an extra-striate visual area (earlier response peak and higher response level at stimulus offset for the striate response) can also be qualitatively captured by cascading the DN model: the first layer takes the stimulus time course as input, and the second layer takes the response output from the first layer as input.

DN is not the only model that captures all four phenomena of neuronal dynamics described in Fig 1. In Fig 7A, we summarize two major classes of models proposed in the literature, some of which are able to qualitatively capture these temporal phenomena, although most have not been fit to and validated against time-resolved neural measurements. These models can roughly be categorized into two classes: RC-circuit inspired models that assume gain control is achieved by a change in response-dependent membrane conductance (red, shown below in Fig 7A), and models that are more computational than mechanistic—they do not assume this particular gain control implementation (blue, shown above in Fig 7A). Whether a model qualitatively captures the temporal phenomena is independent of its type, and comparing models within each type allows us to identify the essential elements required for a model to capture the phenomena.

Within non-RC circuit models (blue), Horiguchi et al. 2009 [11] differs from the other two models in that it lacks a gain control component, albeit it includes exponentiation as a non-linear computation. A consequence is that the Horiguchi et al. model does not capture slow response dynamics at low stimulus contrast (Fig 7B). It does predict an onset transient due to a biphasic impulse response function.

On the other hand, within the RC-circuit model class, Carandini and Heeger 1994 [10], Mikaelian and Simoncelli 2001 [12], and Mante et al 2008 [13] all included a gain control computation. But the Carandini and Heeger model differs from the other two in that its gain control is instantaneous, rather than delayed. Therefore, the Carandini and Heeger model does not capture the response shape (sharp onset transient) for sustained stimuli, assuming a monophasic impulse response function. (This model was developed to fit steady state data, ignoring the initial transient, and hence the omission of the feedback delay was a convenient simplification). From this analysis, we conclude that a history-dependent gain control mechanism is the critical component of a model that successfully captures this set of temporal phenomena. For further physiology evidence supporting the delayed gain control mechanism, see [24]. For why dynamic gain control is critical for removing contrast redundancies in natural images, see [25].

Although multiple models qualitatively capture the temporal phenomena, the DN model is relatively simple to compute, and it is not rooted in the assumption that gain control arises from a change in conductance due to spiking inputs, a mechanism which may not be a sufficient explanation for all forms of normalization [26–28]. (For further accounts of different normalization mechanisms, see Ferster and Miller 2000 [28] and Carandini and Heeger 2012 [8], and for further neural circuit models not covered in Fig 7, see [24, 29, 30]). Every model reflects choices in what aspects of a system to prioritize: one common choice is to emphasize the input-output relationship of a specific information transformation step (computational model), and another is to describe how the transformation is implemented either abstractly or biophysically (algorithmic or mechanistic model). Here, we aimed to develop a compact model that characterizes the functional mapping between stimulus as input and cortical response as output. The cost of this compactness is the omission of biophysical detail of how the model could be implemented by cortical neurons. We chose to model at the current level of abstraction because the model elements can be well constrained by our existing data.

There exist other models of a similar nature constructed for different experiment or measurement types, and models that include gain control without assuming a divisive form. For example, in Tsai et al. 2012 [31], a delayed gain control model (with an exponential delay on the normalization pool) was proposed to capture different frequency responses to different stimulus contrasts in a masking experiment (MEG). The model shares intuition and components with our model. One difference is that the impulse response function in Tsai et al. 2012 was assumed to be a delta function–which is a close approximation for V1 (as evident in their model fits), but not so for later visual areas (as evident in the different τ_1_’s we found in different visual field maps, Fig 4A). A different example is Pillow et al. 2008 [32], in which the model has an LN (linear-nonlinear) computation followed by a recursive subtraction of a spiking-related feedback signal. The subtractive feedback in this model serves the role of gain control. In this case, a subtractive instead of a divisive gain control is sufficient to account for primate retinal ganglion cell (RGC) responses to white noise. A white noise stimulus, given its constant contrast and luminance over time, is not an ideal activator of gain control mechanisms within a cell—gain control adapts the cell’s response to change in environmental inputs. As a consequence, subtractive feedback is likely to be insufficient as a gain control computation for RGCs and cortical cells under natural viewing conditions, in which gain control plays a more crucial role. For more discussion of the difference between subtractive and divisive gain control, see [22] and [33].

Outside the domain of visual cortical computations, dynamic or history-dependent normalization models have been proposed for decision making [34, 35] and attention [36], suggesting that this may be an important general cortical strategy for processing information. One particularly relevant model of this kind was proposed in LoFaro et al. 2014 [34]. The LoFaro et al. model, with its seemingly different input (reward value rather than visual contrast) and structure (differential equations rather than convolution), shares an underlying intuition with our model. The LoFaro et al. model takes the intrinsic value of a choice option as the input/driving signal (analogous to the contrast of a static image in our case), and its gain control signal (normalization signal) depends on an exponentially discounted response history. As our model predicts a transient followed by a sustained response level, the LoFaro et al. model predicts transient and sustained decision activity. One apparent difference between these two models is that the LoFaro et al. model assumes different pools of neurons that carry output and gain control signal, whereas in our model, we make no explicit assumptions of how each computational component maps onto the biophysical constructs. Future work should compare and contrast the equilibrium points of these models at different stimulus inputs and model parameters.

### Related temporal phenomena

The presence of a delayed suppressive signal, as proposed in our DN model, does not preclude the possibility that there are also more rapid suppressive signals. In fact, both psychophysical [37] and neurophysiological studies [38] suggest that local cross-orientation suppression is rapid (maybe even simultaneous with the feedforward inputs) whereas surround suppression is sluggish. Because the stimuli used in our ECoG and fMRI experiments were large, the responses likely included effects of surround suppression. An important goal for future work will be to develop an integrated space-time model to evaluate how the spatial pattern of the stimulus affects the temporal dynamics of the responses.

### Gain control dynamics in membrane-potential measurements

The temporal phenomena studied in this paper mostly pertain to neuronal spiking activities. Some other types of neuronal activities, e.g. membrane potential dynamics, exhibit different shapes and different properties [39, 40]. In Sit et al (2009), Voltage Sensitive Dye Imaging (VSDI), which reflects population membrane potential, has a response time course similar to the Carandini and Heeger 1994 prediction shown in Fig 7A. Moreover, the measured sub-linearity tends to be instantaneous rather than history-dependent, hence a reduced form of the DN model (very small τ_2_) is likely to account for this type of data. Future work explicitly modeling the transformation between membrane potential and spiking in a population of neurons will allow us to connect the two measurement signals.

### Differences across cortical locations

We observed 3 systematic trends across visual cortex: (1) the temporal window length and (2) the degree of normalization increased from V1 to extrastriate areas; (3) the relative sensitivity to transients (reflected in the response to stimulus offsets) increased from fovea to periphery.

#### Temporal window length

The increase in temporal window length was systematic but small, increasing by about 30% from V1 to the anterior maps just beyond V3. Qualitatively, this is similar to the increase in spatial receptive field size across the cortical hierarchy, but the differences in spatial receptive fields are larger: receptive field size more than doubles from V1 to V3, and increases by at least 4 times from V1 to V4, measured with either single units [41–44] or fMRI [17, 45, 46]. Other studies have also found a hierarchy of temporal processing human and macaque cortex. Hasson et al [47] quantified temporal windows in human based on the response reliability to scrambled movie segments. They found evidence for very long temporal windows in high level areas such as the superior temporal sulcus (> 10 s). These longer windows compared to our results are likely a result of both the brain areas studied and the methods. Temporal cortical hierarchies have also been measured [48] and modeled [30] in macaque. Murray and colleagues [48] found that the time-scale of an area while the animal was at rest (time-constant of the temporal autocorrelation function) was relatively short in early sensory areas (~100 ms or less) and longer in higher level association areas (up to ~300 ms), more commensurate with our results. In our study, we modeled all areas with the same model form, and found that the parameters changed across areas. The model could be re-expressed as a cascade, in which later areas go through more iterations than earlier areas. We show by simulation that a cascaded DN model produces a qualitatively similar pattern of results to those we observe in higher cortical areas (S4B Fig). In their model, Chaudhuri and colleagues [30] also capture the hierarchy of temporal scales, although they do not include normalization and do not account for the shape of the temporal response, such as the transient response at stimulus onset.

#### Normalization

In addition to the increasing temporal summation window length, we also found an increasing extent of normalization from early to late visual areas. This gradation of neuronal adaptation levels is consistent with previous results showing that the anterior visual field maps sum more compressively in time [4]. The combination of longer temporal windows and more adaptation may together cause responses in later areas to show less sensitivity (more tolerance) to changes in stimulus duration or timing, paralleling the greater tolerance for changes in stimulus size and position [17]. This pattern is also consistent with the observation that activity in early visual areas tends to stay elevated for longer durations while activity in category-selective ventral temporal areas tends to decline more rapidly following stimulus onset [49].

Note that the difference in response profiles (earlier response peak and higher response level at stimulus offset) between striate and extra-striate areas can also be qualitatively captured by a cascaded DN model, as compared to a one-stage DN model implemented with different parameters (Fig 7C). The first layer of the cascaded DN model takes a stimulus contrast time course as input, and the second layer of the model takes the output response from the first layer as input, and produces another time course response as output. The second layer of the cascaded DN model shares the same parameters as the first layer. This analysis indicates one possible explanation for the different response profiles we measured across the visual areas: the delay in time to peak and the reduced response amplitude at stimulus offset roughly reflects the number of stages in the cascaded processing.

#### Stimulus offset responses

We found that temporal dynamics varied not only between maps but also within maps. Specifically, within V1-V3, peripheral response time courses measured by ECoG tended to exhibit large transient at stimulus offset. As a consequence, the peripheral responses, dominated by the onset and offset transients, are more sensitive to changes in stimulus contrast, whereas the foveal responses are more sensitive to the stimulus duration. It is likely that these differences start to emerge early in visual processing. For example, the ratio of parasol to midget cells is higher in the periphery than the fovea, contributing to higher sensitivity to transients [50]. Even within a cell class, the midget ganglion cells show faster dynamics in the periphery than the fovea [51]. The greater sensitivity to transients in the periphery and sustained signals in the fovea likely reflects differences in information processing across the visual field: the periphery plays an important role in exogenous attention (responding to changes in the environment), whereas the fovea is involved object recognition and appearance.

### Generalization and future directions

Our model establishes baseline performance by demonstrating explanations of several important phenomena obtained for static, large-field images over a few hundred milliseconds. This type of stimuli is well matched to many natural tasks such as scene exploration and reading, in which fixations of (mostly) static images alternate with saccades, at approximately this time scale [52]. Moreover, the model serves as a valuable platform for further development to account for other stimulus manipulations and task conditions. For example, sustained attention to the stimulus [19], presence of a surround [53], non-separable spatiotemporal patterns (motion), and stimulus history of many seconds or more [54], can all affect the time course of the response.

## Methods

### Participants

ECoG data were re-analyzed from prior work [16]. As reported previously, those data were measured from 2 participants who were implanted with subdural electrodes for clinical purposes. The participants gave informed consent to participate in the study and the study was approved by the Stanford University IRB.

Functional MRI data was re-analyzed from prior work [4]. As we reported previously, these data came from 6 experienced fMRI participants (2 males and 4 females, age range 21–48 years, mean age 31 years) and were collected at the Center for Brain Imaging at New York University. The experimental protocol was approved by the University Committee on Activities Involving Human Subjects at New York University, and informed written consent was obtained from all participants before the study.

### ECoG Procedure

#### Preprocessing

The data were pre-processed as in [16]. In brief, electrodes that had large artifacts or epileptic activity, as identified by the neurologist, were excluded from analysis. From the remaining electrodes, we re-referenced the time series to the common average, and then down sampled the data from the recorded frequency of either 3052 or 1528 Hz to 1,000 Hz.

#### Trial structure

At the beginning of each 1-second trial, a large field (22°) noise image was randomly selected from one of 8 image classes. Several of these image classes were chosen for studying gamma oscillations in the original paper, which differs from the purpose of the current study. For this study, we analyzed data from the noise image classes only (3 of the 8 image classes): white, pink, and brown noise (amplitude spectra proportional to 1/*f*^0^, 1/*f*^1^, 1/*f*^2^). Noise images tend to induce a broad gamma band amplitude increase only in field potential recordings in the visual cortex, which is thought to correlate with increased spike rate and BOLD [55]. Each image was presented for 500 ms followed by a 500ms blank. We analyzed data in 1200ms epochs, beginning 200 ms prior to stimulus onset and ending 500 ms after stimulus offset.

#### Broadband envelope

We extracted the broadband component of the ECoG signal for model fitting and other analyses. The broadband signal is thought to be correlated with local spiking activities: the broadband signal correlates with multi-unit activities near electrodes [56] and with BOLD fMRI response [57, 58].

A number of steps were taken to extract time-varying broadband signals, which can be summarized as the power of the envelope of band-pass filtered ECoG voltage time courses. First, the raw ECoG time courses were band-pass filtered in several bands within the range between 70 and 210 Hz. Below 70 Hz, signals are more likely to be influenced by low frequency cortical rhythms and other processes and may contaminate the broadband estimation [59]. Above 210 Hz, amplifier noise reduces SNR and therefore we set the amplifier-dependent ceiling for the analysis.

Instead of band-pass filtering the entire voltage time course with a single filter, we took ten 10-Hz bins from 70 to 210 Hz (70–80 Hz, 80–90 Hz, skipping 60 Hz line noise and its harmonics) using Butterworth filter (passband ripples < 3 dB, stopband attenuation 60 dB). 10 Hz bins were chosen because we estimated that we need at least 10 Hz bins to capture the sharp transients in the spike rates, and bandwidths broader than 10 Hz do not affect the estimated shapes of the spike rate transients any further. The reason for multiple bands is because the power in field potentials declines with frequency; if we computed the envelope of a single large pass band (say, 70–210 Hz), it would be dominated by signals near 70 Hz. After extracting the multiple bands, their time series were combined by computing a geometric mean, which ensures that the high frequency bands (low power) still contribute.

#### Broadband units

To convert the unit of the time-varying broadband to percent signal change in each electrode, we first averaged each broadband time series across epochs. We defined the first 200 ms prior to stimulus onset as the baseline period for the epoch-averaged time course, then we computed the percent signal change by dividing the entire 1200ms time course point-wise by the average of the baseline. To equalize the baseline across electrodes, we subtracted the baseline average from the entire time-course, so each electrode has trial-averaged baseline 0.

#### Electrode selection

We first selected all electrodes located in identifiable visual areas based on separate retinotopy scans. Among these location-identifiable electrodes, we only chose the electrodes that satisfy the following two criteria for further analysis: 1. electrodes whose trial-averaged broadband response during the stimulus on period (500 ms) is greater than the baseline period on average; 2. electrodes whose maximal trial-averaged broadband response is greater than 150% of the pre-normalized baseline average (see *Broadband units*). (See *dn_chooseElectrodes*.*m*).

#### Foveal versus peripheral electrodes

Based on the retinotopy analysis, we separated the electrodes within V1-V3 into three eccentricity bins (<5, 5–10, >10 degrees) based on their estimated receptive field centers.

### Single- and Multi-unit Procedure

#### Single-units preprocessing

We re-analyzed two macaque single-unit data sets from Albrecht et al. 2002. The first data set consists of trial-averaged PSTH from 12 complex cells in V1 (S2A Fig), and each PSTH represents a distinct type of response shape (Albrecht et al. 2002, their Fig 4). The stimulus for this data set is a large field spatial grating presented over 10 different contrasts and 9 spatial phases. Each PSTH in the data set is the response averaged across 40 repetitions of each pair of contrast and spatial phase combination. To generate the average single-unit time course in S2 Fig (panel A), we first duplicated each PSTH *n* times, with *n* being the number of cells within each shape category (see Albrecht et al. 2002 Fig 4). We then bootstrapped over this expanded cell set by randomly sampling (100 times) with replacement. Finally, the DN model was fitted to the average of the bootstraps (see DN Model Fit).

The second data set (Albrecht et al. 2002 Fig 1) consists of three cells’ responses to a 200 ms presentation of a large field stationary grating (8 different spatial phases) at 10 linearly spaced (0–90%) contrast levels (Fig 6).

#### Multi-units preprocessing

We re-analyzed the data correspond to the “Contextual modulation” experiment in [19]. The stimulus used in the experiment consists of one (stationary) spatial grating restricted to a circular patch, and the rest of the screen is filled with another grating that is of the same spatial frequency, but the same or different orientation and phase. Each grating is presented at 80% Michelson contrast, and with a spatial frequency 1 cycle/degree. In each trial, the stimulus is on for 500ms before the screen returned to neutral gray. For our purpose, we pooled signals across all trial types (S2A Fig).

MUA and LFP extractions are exactly the same as described in [19]. We fitted the DN model to the trial-averaged MUA signals, and the time-varying broadband envelopes within the LFP signal. The broadband extraction process of the LFP signal is the same as that described in *Broadband envelope* under the ECoG Procedure, except for one minor difference: 1. We band-pass filtered the time series in ten 10-Hz bins from 85 Hz to 175 Hz (excluding harmonics of 50Hz line noise), instead of from 70 to 210 Hz as in the ECoG data. We chose a higher starting frequency (85 Hz) here because grating stimuli tend to induce an oscillatory signal within 30–80 Hz range measured on the visual cortex, and we chose a lower ending frequency (175 Hz) because the sampling rate here is lower than the ECoG data. (See *dn_analyzeMultiDataTypes*.*m*).

### Models of temporal dynamics

#### Models

*Delayed Normalization (DN) model*. The DN model takes the contrast time course (*T*input) of a stimulus presentation as input, and the model consists of a linear convolution, an exponentiated rectification, and a divisive normalization stage.

First, for the linear stage, a weighted difference between two gamma functions was used as the form of the impulse response function. Each gamma function is simplified from the following equation [60]:

$$h(\tau_{1})=\frac{{\left(\frac{t}{\tau_{1}}\right)}^{\left(m-1\right)}e^{-(\frac{t}{\tau_{1}})}}{\tau_{1}(m-1)!}$$

to

$$h(\tau_{1})={te}^{-t/\tau_{1}}$$

by assuming *m* = 2. We further normalize each *h*(*τ*_1_) by its sum. We do not allow the *m* parameter to vary because our current data does not have the power to distinguish between the change in *m* and the change in *τ*_1_ in the normalized equation.

The function peaks when *t = τ*_1_. To account for the post-stimulus transient response in some of the data, we used a weighted difference of two such gamma functions as the impulse response function in the paper:

$$h1(\tau_{1},w)={te}^{-t/\tau_{1}}-{te}^{-t/{1.5\tau }_{1}}$$

Here, we assumed the peak timing of the second gamma function to be 1.5 times the first one, and we vary *w* only in Fig 4B, S3 and S4A Figs because we do not observe prominent offset transient response in ECoG time courses averaged across electrodes within an ROI, or in the single unit spiking time courses. For Figs 3 and 4A, we fixed *w* to be 0, so only a single gamma function is used as the impulse response function. The linear response is computed by convolving the impulses response function, with the contrast time course of a stimulus presentation:

$$R_{L}=T_{input}*h1(\tau_{1},w)$$

The exponential rectification, following the linear computation, is of the form

$$R_{LN}={{|R}_{L}|}^{n}$$

The exponential rectification may be thought of a correlate of the non-linearity resulting from thresholded spiking.

The purpose of the exponential rectification is to capture the non-linearity resulted from thresholded spiking. The last computation in the model is a dynamic, or history-dependent divisive normalization. The numerator of the division is *R*_*LN*_. The denominator consists of two components. The first component is a semisaturation constant *σ*, raised to the same power *n* as the numerator. The semisaturation term prevents the equation from going undefined when the input stimulus has 0 contrast at every time point.

The second term in the denominator represents the response dynamics of the normalization pool. The term is a low-pass filtered version of the numerator, and the low-pass filter is implemented by an exponential decay, parameterized by τ_2_:

$$h2(\tau_{2})=e^{-t/\tau 2}$$

Over all, the divisive normalization can be represented as.

$$R_{DN}=\frac{{{|}_{LN}|}^{n}}{\sigma^{n}+{{|R}_{LN}|*h2(\tau_{2})]}^{n}}$$

To fit the first DN model to the time series data (SUA, MUA, LFP broadband and ECoG broadband), we vary all four model parameters (*τ*_1_, *τ*_2_, *n*, *σ*) together with two nuisance parameters. The first nuisance parameter represents a delay that accounts for the time elapsed between stimulus onset and response onset; the second nuisance parameter scales the predicted model output to the same range as the measured signals. (See *dn_DNmodel*.*m*).

*Two temporal channels model* (S4 Fig, Horiguchi et al. 2009; Stigliani et al 2017). The model consists of a weighted sum of two components–the components are interpreted as the output of a sustained and a transient temporal channel. The output of the sustained component follows a linear computation, and the output of the transient channel follows a sub-linear computation:

$$R_{2TC}=a[IRF_{sustained}*T_{input}]+b{\left[IRF_{transient}*T_{input}\right]}^{2}$$

A higher weight of the transient component leads to a higher degree of the offset transient response, and a higher weight of the sustained component leads to a higher level of the sustained response. (See *dn_2Chansmodel*.*m*) This model was inspired in part by two-temporal models used to explain psychophysical performance (e.g. [61–63]) and electrophysiology (e.g. [64]).

*Compressive Temporal Summation* (S4 Fig, [4]). The compressive temporal summation (CTS) model is similar to the DN model except that the normalization is instantaneous instead of delayed as in the DN.

$$R_{CTS}=\frac{{R_{linear}}^{n}}{{\sigma^{n}+R_{linear}}^{n}}$$

For simplicity, we assumed n = 2 when fitting this model to the ECoG time course in S4 Fig. (See *dn_simpNormModel*.*m*).

*Cascaded DN model* (S4 Fig). When fitting the DN model to the ECoG broadband time courses, we assumed that for each visual area, the DN model takes a stimulus as input and produces a response time course as output. Alternatively, we also illustrate the behavior of a two-stage cascade model: we used the output of the V1 model as the input to a second, identical model, and show that this produces responses qualitatively similar to V3AB.

#### Parameter estimation

*DN model for ECoG*. We used a two-stage approach to fitting the DN model, first to obtain seeds (grid fit) and then to estimate parameters (search fit). For the grid fit, we computed model predictions to the 500 ms stimulus for 10,000 combinations of τ_1_, τ_2_, *n*, and σ (τ_1_: [0.07, 1], τ_2_: [0.07, 1], *n*: [1, 6], σ: [0.01, 0.5], each parameter in the range with 10 equal steps). Using linear regression on the data time course, we derived the gain factor, *g*, and the variance explained for each of the 1,000 predicted time series. For each bootstrapped response time course in each ROI, the set of parameters that generated the highest variance explained was used as the seed for the search fit. (See *dn_gridFit*.*m)*

For the search fit, we used a bounded nonlinear search algorithm in MATLAB (*fminsearchbnd*.*m*), run once per ROI per bootstrap. The search finds the parameters that minimize the squared error between the predicted and the measured time broadband course. The lower bound used for the search fit was [0.07, 0.07, 1, 0.01, 0.0001] for τ_1_, τ_2_, *n*, σ and a shift parameter that accounts for the delay between stimulus onset and response onset. The upper bound used for the search fit was [1, 1, 6, 0.5, 0.1]. In principle, the delay parameter is important, since the time at which the signal from the stimulus reaches cortex is delayed, and the delay varies across visual field maps, and could be as high as 50–150 ms. However, the impulse response function includes a slow ramp, and the broadband envelope extraction contains a small amount of blur. Hence in practice, the shifts were quite small (< 10 ms), and not informative about the latency of neuronal response. To summarize the fit, we plotted the mean of the predicted time course across bootstraps and the standard deviation at each time point as the confidence interval. (See *dn_fineFit*.*m*).

*DN model for single-unit data with variable stimulus contrast (*Fig 6). We fit the DN model to three cells’ response time course to a 200-ms stimulus contrast increment at 10 different contrast levels (0% − 90% contrast with 10 steps of equal increment). We fit one set of DN parameters to all 10 response time courses for each cell by minimizing the squared error between the data and prediction. We seeded the search fit for the first two cells with [0.1, 0.1, 2, 0.2, 0.03], and the last cell with [0.1, 0.1, 3, 0.1, 0.04] (τ_1_, τ_2_, *n*, σ, and a shift parameter). Then we used *fminsearch*.*m* in MATLAB for the search fit. (See *dn_mkFigure_fitDN2ContrastSUA*.*m*).

*DN model for fMRI BOLD amplitude* (Fig 5). To predict the fMRI response from the DN model, we used the parameters fitted from ECoG data for each electrode (S3 Fig), took the median of each parameter within each ROI, and generated a neuronal time course for each of the 13 distinct temporal profiles from the fMRI experiment. Then we summed each predicted time course, and finally scaled the sum by a gain factor, *g*. The only free parameter was the gain factor. (See *dn_fitDNECoG2fMRI*.*m*)

$${BOLD}_{DN}=g[\sum R_{DN}]+e$$

*Biphasic DN model fit to ECoG broadband* (Fig 4B). To fit the DN model with a biphasic IRF to the broadband time course estimated for individual ECoG electrodes, we varied five model parameters together: τ_1_, *w*, τ_2_, σ, and a shift parameter. “*w*” is the weight of the negative pulse in the IRF. The length of the second pulse, τ_2_, was assumed to be 1.5 times τ_1_, and *n* was assumed to be 2. For each electrode, we generated four predictions from these four sets of parameters first: [0.02, 0.8, 0.15, 0.1, 0.05]; [0.03, 0.8, 0.1, 0.2, 0.05]; [0.02, 0.4, 0.15, 0.1, 0.05]; [0.03, 0.4, 0.1, 0.2, 0.05]. These parameter sets differ in the extent of normalization and the extent of the post-stimulus transient response. We picked the parameter set that generated the highest variance explained for each electrode, and used the set as the seed for a further search fit. (See *dn_mkFigure_bidnFit2ECoG*.*m*).

#### Model accuracy

Throughout the paper, we summarized model accuracy as the variance explained, *r*^*2*^, the square of the Pearson-correlation coefficient *r*.

### Public data sets and software code

To ensure that our computational methods are reproducible, all data and all software will be made publicly available via an Open Science Framework web site, https://osf.io/z7e3t/. The software repository will include scripts of the form *dn_mkFigure2*.*m* to reproduce Fig 2, etc., as in prior publications [4].

## Supporting information

S1 Fig How each parameter in the DN model affects the model prediction. Here we explore how different DN model parameters affect the model predictions to two 500ms stimulus time courses (1 and 0.3 in contrast respectively). The black curve in each panel indicates the predicted response time course to the high contrast stimulus (at a chosen set of parameters), and the gray curve indicates response to the low contrast stimulus. The range of values we sweep across for each parameter is the range of values we used for the grid search step to fit each model parameter. In general, the DN model predicts an initial transient response followed by a decay. The width of the initial transient increases with increase in *τ*_1_ value. Because the model parameters interact with each other, the width of the initial transient depends also on the value of *n* and *σ*. Furthermore, *n* and *σ* controls the decay rate of the transient response. *τ*_2_ controls how smooth the transient response decays, and *w* controls the extent of the post-stimulus transient. The parameter trade-off could potentially be resolved by comparing model predicted time course to different stimulus contrasts: for example, changing parameter *n* scales a high contrast response time course to a different extent to predict for the low contrast response without changing the response shape, whereas changing *σ* changes predicted shape for the low contrast response without changing much of its relative scale to the high contrast response. Related to Fig 2.
(TIF)

S2 Fig Individual electrode responses. The plots show the ECoG broadband time course in individual electrodes from ECoG subject S1, averaged across 90 trials (30 repeats each of three stimulus types). Each row shows electrodes from one ROI. Some electrodes (e.g., 74) are in two rows, since the electrode was near an ROI boundary. The plots are color coded by eccentricity bin (0–5°, 5–10, >10°). The pRF location was based on a separate ECoG pRF data set published previously (Winawer et al., 2013). The two mesh images show a magnified view of S1’s right occipital lobe, exposing the medial surface (left) and lateral surface (right). Insets show the zoomed-out view of the cortical mesh. Related to Figs 3 and 4.
(TIF)

S3 Fig Cross-validated predictions. Cross-validation over trials. During the experiment, the subject was presented with large field white, pink, and brown noise stimuli, and each image class was repeated over 30 times. Each electrode’s response to different image class was slightly different (e.g. a foveal electrode responded with higher amplitude to white noise compared to brown noise stimuli), and the DN model does not have a spatial component to capture such differences. To discount such differences when cross-validate, we took each “trial” as the average response over one repeat of white, pink, and brown noise images. The black curves were the left-out response, and the red was the DN prediction based on the other 29 “trials.” Each row represents a different ROI, and each column represents a left-out trial. Cross-validation over electrodes. The black curves are the trial-averaged responses from the left-out electrodes, and the red curves are the DN model prediction based on the rest of the electrodes within an ROI. Each row represents an ROI, and each column represents a left-out electrode. Related to Fig 3.
(TIF)

S4 Fig Response reduction for prolonged stimuli. (A) Response time courses from 3 different recording methods are shown. In each plot, the data are in black (±1 sem in gray) and the DN model fit in red. Left: single unit spike rates, averaged across neurons in macaque V1. Middle: Multiunit spike rates from human V2/V3. Right: High frequency broadband power (LFP) from human V2/V3. (B) DN model parameters from human ECoG. The model parameters in each of 4 ROIs are shown for the data plotted in the main text (Fig 3A). (C) ECoG broadband responses in 3 ROIs from subject S2. Plotting conventions as in Fig 3. Related to Figs 3 and 4.
(TIF)

S5 Fig Effects of eccentricity and contrast on temporal dynamics. Individual electrode time courses and DN model fits in V1-V3. The background color indicates the eccentricity bins: 0°-5° (red), 5°-10° (purple), and >10° (green). There is a general tendency toward greater offset responses in more peripheral electrodes. Related to Fig 4.
(TIF)
