# Supplementary figures and images for: Predicting neuronal dynamics with a delayed gain control model
Source: PLoS Comput Biol. 2019 Nov 20;15(11):e1007484. doi: 10.1371/journal.pcbi.1007484 (PMC6892546; doi:10.1371/journal.pcbi.1007484)

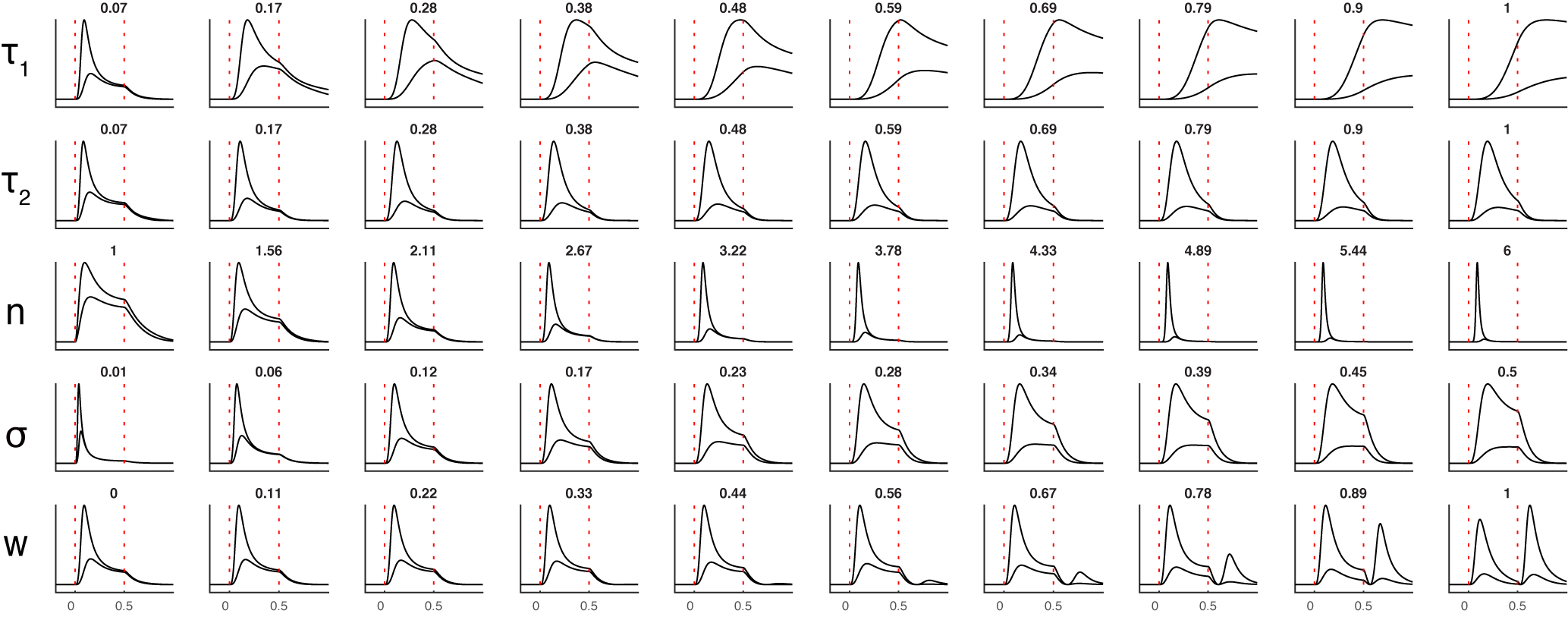

Supplement: S1 Fig — Here we explore how different DN model parameters affect the model predictions to two 500ms stimulus time courses (1 and 0.3 in contrast respectively). The black curve in each panel indicates the predicted response time course to the high contrast stimulus (at a chosen set of parameters), and the gray curve indicates response to the low contrast stimulus. The range of values we sweep across for each parameter is the range of values we used for the grid search step to fit each model parameter. In general, the DN model predicts an initial transient response followed by a decay. The width of the initial transient increases with increase in τ1 value. Because the model parameters interact with each other, the width of the initial transient depends also on the value of n and σ. Furthermore, n and σ controls the decay rate of the transient response. τ2 controls how smooth the transient response decays, and w controls the extent of the post-stimulus transient. The parameter trade-off could potentially be resolved by comparing model predicted time course to different stimulus contrasts: for example, changing parameter n scales a high contrast response time course to a different extent to predict for the low contrast response without changing the response shape, whereas changing σ changes predicted shape for the low contrast response without changing much of its relative scale to the high contrast response. Related to Fig 2. (TIF) [file pcbi.1007484.s001.tif]

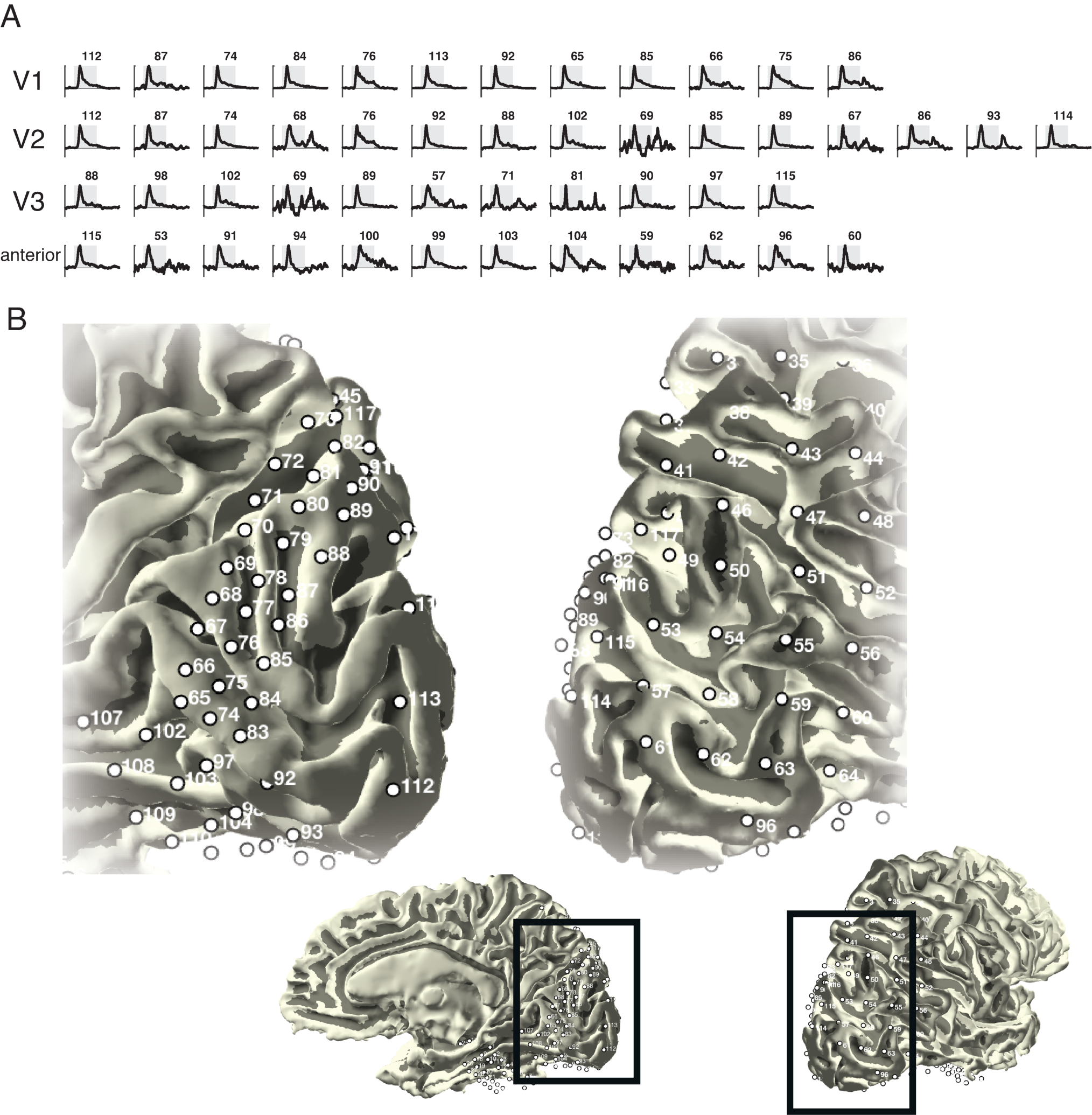

Supplement: S2 Fig — The plots show the ECoG broadband time course in individual electrodes from ECoG subject S1, averaged across 90 trials (30 repeats each of three stimulus types). Each row shows electrodes from one ROI. Some electrodes (e.g., 74) are in two rows, since the electrode was near an ROI boundary. The plots are color coded by eccentricity bin (0–5°, 5–10, >10°). The pRF location was based on a separate ECoG pRF data set published previously (Winawer et al., 2013). The two mesh images show a magnified view of S1’s right occipital lobe, exposing the medial surface (left) and lateral surface (right). Insets show the zoomed-out view of the cortical mesh. Related to Figs 3 and 4. (TIF) [file pcbi.1007484.s002.tif]

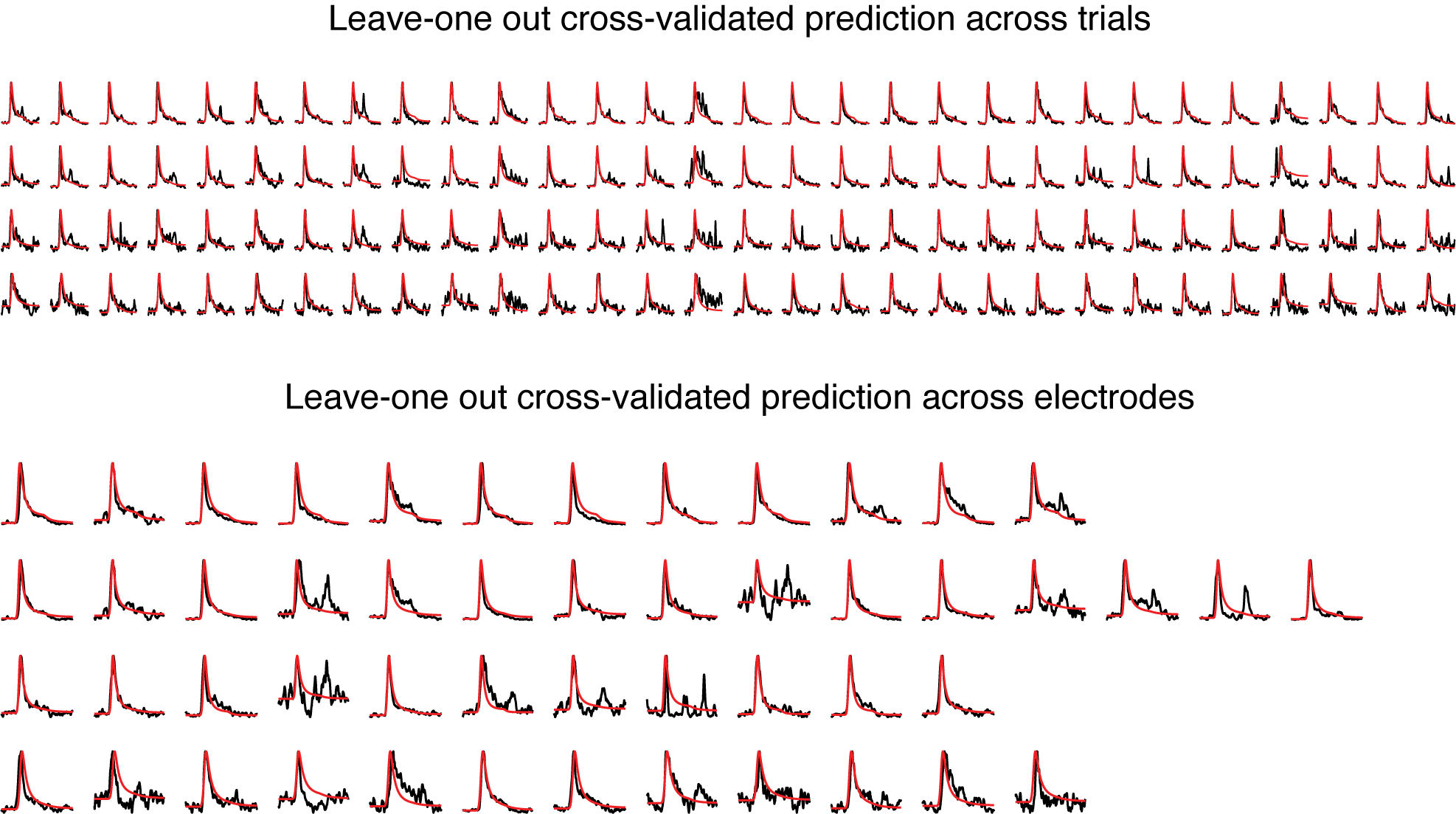

Supplement: S3 Fig — Cross-validation over trials. During the experiment, the subject was presented with large field white, pink, and brown noise stimuli, and each image class was repeated over 30 times. Each electrode’s response to different image class was slightly different (e.g. a foveal electrode responded with higher amplitude to white noise compared to brown noise stimuli), and the DN model does not have a spatial component to capture such differences. To discount such differences when cross-validate, we took each “trial” as the average response over one repeat of white, pink, and brown noise images. The black curves were the left-out response, and the red was the DN prediction based on the other 29 “trials.” Each row represents a different ROI, and each column represents a left-out trial. Cross-validation over electrodes. The black curves are the trial-averaged responses from the left-out electrodes, and the red curves are the DN model prediction based on the rest of the electrodes within an ROI. Each row represents an ROI, and each column represents a left-out electrode. Related to Fig 3. (TIF) [file pcbi.1007484.s003.tif]

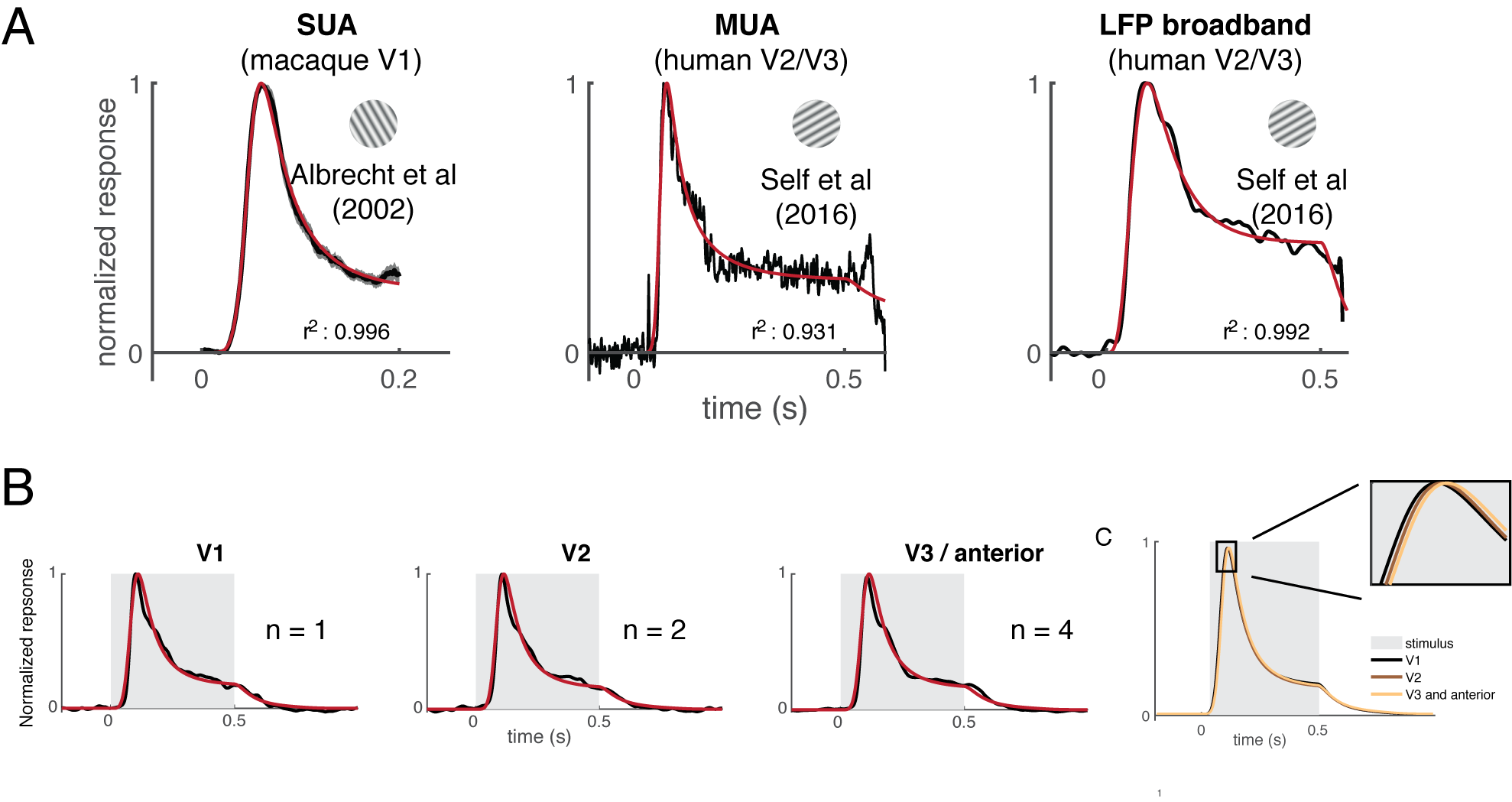

Supplement: S4 Fig — (A) Response time courses from 3 different recording methods are shown. In each plot, the data are in black (±1 sem in gray) and the DN model fit in red. Left: single unit spike rates, averaged across neurons in macaque V1. Middle: Multiunit spike rates from human V2/V3. Right: High frequency broadband power (LFP) from human V2/V3. (B) DN model parameters from human ECoG. The model parameters in each of 4 ROIs are shown for the data plotted in the main text (Fig 3A). (C) ECoG broadband responses in 3 ROIs from subject S2. Plotting conventions as in Fig 3. Related to Figs 3 and 4. (TIF) [file pcbi.1007484.s004.tif]

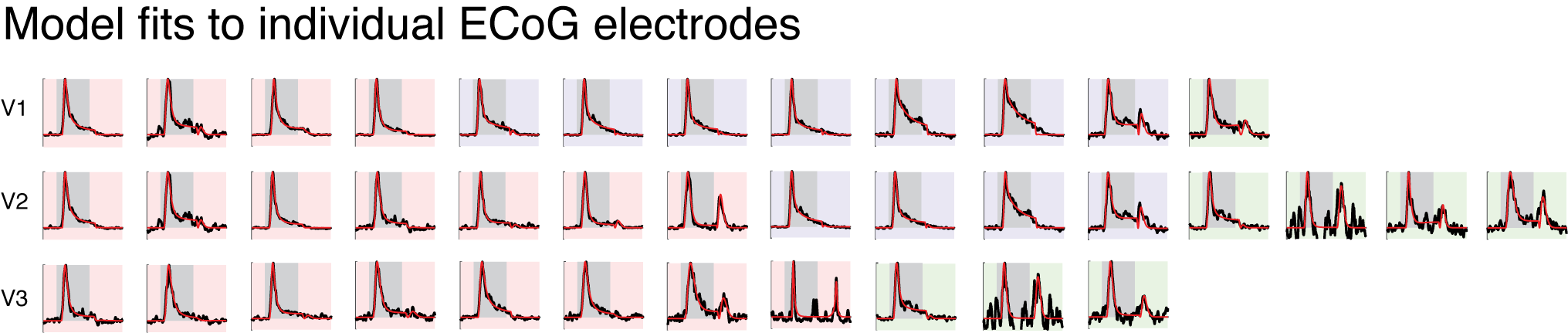

Supplement: S5 Fig — Individual electrode time courses and DN model fits in V1-V3. The background color indicates the eccentricity bins: 0°-5° (red), 5°-10° (purple), and >10° (green). There is a general tendency toward greater offset responses in more peripheral electrodes. Related to Fig 4. (TIF) [file pcbi.1007484.s005.tif]
